# *N*,*N*-Bis(2-ethylhexyl)-((1,2,4-triazol-1-yl)methyl)amin: MAK-Begründung, Nachtrag

**DOI:** 10.34865/mb9127304ksd11_1ad

**Published:** 2026-03-30

**Authors:** Andrea Hartwig, MAK Commission

**Affiliations:** 1 Institut für Angewandte Biowissenschaften, Abteilung Lebensmittelchemie und Toxikologie, Karlsruher Institut für Technologie (KIT) Adenauerring 20a, Geb. 50.41 76131 Karlsruhe Deutschland; 2 Ständige Senatskommission zur Prüfung gesundheitsschädlicher Arbeitsstoffe, Deutsche Forschungsgemeinschaft, Kennedyallee 40, 53175 Bonn, Deutschland. Weitere Informationen: Ständige Senatskommission zur Prüfung gesundheitsschädlicher Arbeitsstoffe | DFG

**Keywords:** N,N-Bis(2-ethylhexyl)-((1,2,4-triazol-1-yl)methyl)amin, Nase, oberer Atemtrakt, Kanzerogenität, Reizwirkung, Sensibilisierung, Keimzellmutagenität, Formaldehyabspalter, Strukturanalogie

## Abstract

The German Senate Commission for the Investigation of Health Hazards of Chemical Compounds in the Work Area (MAK Commission) has re-evaluated *N*,*N*-bis(2-ethylhexyl)-((1,2,4-triazol-1-yl)methyl)amine [91273-04-0] with regard to its carcinogenicity and germ cell mutagenicity classification, its ability to be absorbed through the skin, its sensitization potential and whether an occupational exposure limit value (maximum concentration at the workplace, MAK value) can be derived. Relevant studies were identified from a literature search. *N*,*N*-Bis(2-ethylhexyl)-((1,2,4-triazol-1-yl)methyl)amine is corrosive to the skin of rabbits. The substance is a formaldehyde releaser and is expected to undergo rapid hydrolysis in aqueous solution. For this reason, the effects are attributed to the hydrolysis products formaldehyde, bis(2-ethylhexylamine) and 1H-1,2,4-triazol. There are no studies available that investigated the carcinogenicity, toxicity and genotoxic potential of *N*,*N*-bis(2-ethylhexyl)-((1,2,4-triazol-1-yl)methyl)amine in the upper respiratory tract or nose, which are the likely target organs. Formaldehyde was classified in Carcinogen Category 4 because it induces tumours in nasal tissue at concentrations that exceed its detoxification capacity. As a formaldehyde releaser, *N*,*N*-bis(2-ethylhexyl)-((1,2,4-triazol-1-yl)methyl)amine could likewise be classified in Carcinogen Category 4. However, because it is not possible to derive a MAK value for *N*,*N*-bis(2-ethylhexyl)-((1,2,4-triazol-1-yl)methyl)amine, the substance has been assigned to Carcinogen Category 2 with the footnote “Prerequisite for Category 4 in principle fulfilled, but insufficient data available for the establishment of a MAK or BAT value”. As there are no data available for the systemic bioavailability of *N*,*N*-bis(2-ethylhexyl)-((1,2,4-triazol-1-yl)methyl)amine and the formaldehyde released by hydrolysis in tissues, there is no experimental evidence that the formaldehyde reaches the germ cells. Therefore, *N*,*N*-bis(2-ethylhexyl)-((1,2,4-triazol-1-yl)methyl)amine has been classified in Category 3 B for germ cell mutagens. Data on the sensitizing effects in humans are not available. However, maximization tests in guinea pigs revealed a skin sensitizing potential, and the substance has therefore been designated with “Sh”. Skin contact is not expected to contribute significantly to systemic toxicity.

**Table:** 

**MAK-Wert **	**–**
**Spitzenbegrenzung **	**–**
	
**Hautresorption**	**–**
**Sensibilisierende Wirkung (2016)**	**Sh **
**Krebserzeugende Wirkung (2025)**	**Kategorie 2** ^a)^
**Fruchtschädigende Wirkung**	**–**
**Keimzellmutagene Wirkung (2025)**	**Kategorie 3 B**
	
**EKA**	**–**
	
Synonyma	1-(*N*,*N*-Bis(2-ethylhexyl)aminomethyl)-1,2,4-triazol 2-Ethyl-*N*-(2-ethylhexyl)-*N*-(1,2,4-triazol-1-ylmethyl)hexan-1-amin
CAS-Nr.	91273-04-0
Dampfdruck bei 20 bis 25 °C	7,1 × 10^–5^ hPa (ECHA 2021; Hartwig und MAK Commission 2017)
Hydrolysestabilität	hydrolysiert bei Kontakt mit Wasser fast sofort; die identifizierten Hydrolyseprodukte sind Formaldehyd, Bis(2-ethylhexylamin) und 1H-1,2,4-Triazol (k. w. A.; ECHA 2021)

^a)^ Voraussetzung für Kategorie 4 prinzipiell erfüllt, aber Daten für MAK- oder BAT-Wert-Ableitung nicht ausreichend

Hinweis: Formaldehydabspalter

Es liegt bereits eine Begründung (Hartwig und MAK Commission 2017) vor. In diesem Nachtrag erfolgt eine Neubewertung der Daten zur Formaldehydabspaltung nach aktuellem Vorgehen der Kommission (DFG 2025). Unter diesem Aspekt werden in diesem Nachtrag die bewertungsrelevanten Endpunkte reevaluiert.

## Allgemeiner Wirkungscharakter

Der vermutlich kritische Effekt von *N*,*N*-Bis(2-ethylhexyl)-((1,2,4-triazol-1-yl)methyl)amin ist die kanzerogene und reizende Wirkung von freigesetztem Formaldehyd am Atemtrakt. *N*,*N*-Bis(2-ethylhexyl)-((1,2,4-triazol-1-yl)methyl)amin wirkt hautsensibilisierend und an der Haut von Kaninchen ätzend. Für *N*,*N*-Bis(2-ethylhexyl)-((1,2,4-triazol-1-yl)methyl)amin ergibt sich kein Verdacht auf eine genotoxische Wirkung in somatischen Zellen. Daten zur wiederholten inhalativen Exposition, Keimzellmutagenität und Kanzerogenität liegen nicht vor.

## Wirkungsmechanismus

*N*,*N*-Bis(2-ethylhexyl)-((1,2,4-triazol-1-yl)methyl)amin ist ein Formaldehydabspalter.

## Toxikokinetik und Metabolismus

*N*,*N*-Bis(2-ethylhexyl)-((1,2,4-triazol-1-yl)methyl)amin hydrolysiert in Wasser sofort zu Bis(2-ethylhexylamin) (CAS-Nr. 106-20-7, Wasserlöslichkeit 0,014 g/l, log K_OW_ 7,3; ECHA 2023), 1H-1,2,4-Triazol (CAS-Nr. 288-88-0, Molmasse 69,07 g/mol, Wasserlöslichkeit 700 g/l, log K_OW_ –0,76 (berechnet); ECHA 2022 a) und Formaldehyd (Molmasse 30 g/mol, Wasserlöslichkeit 550 g/l, log K_OW_ 0,35; ECHA 2022 b). Wegen der sofortigen Hydrolyse ist eine Messung der Wasserlöslichkeit und damit eine Berechnung der Hautresorption des Stoffs mit den Modellen nicht möglich. Da der Stoff an der Haut ätzend wirkt (Skin Corr 1B; ECHA 2023), und nach CLP-Verordnung eine 0,5%ige Lösung nicht als hautreizend eingestuft werden muss, wird für die Bewertung der Hautresorption von den Hydrolyseprodukten bei dieser Konzentration ausgegangen.

Bis(2-ethylhexylamin) ist ätzend und es gibt keine Studien zur Hautresorption. Eine Berechnung mit den Modellen ist nicht möglich, da der log K_OW_ > 6 ist und damit außerhalb des Gültigkeitsbereichs der Modelle liegt.

Für festes 1H-1,2,4-Triazol wurde in einem In-vitro-Test bei 24-stündiger Applikation von 5 mg auf 1 cm^2^ exzidierte Humanhaut eine in und durch die Haut penetrierte Menge von 132 µg/cm^2^ (2,7 % der applizierten Menge) nach 24 Stunden bestimmt (ECHA 2022 a). Der Flux entspricht damit 5,5 µg/cm^2^ und Stunde und bei 2000 cm^2^ exponierter Hautfläche beträgt die Aufnahmemenge 11 mg. Für eine 0,5%ige nicht mehr reizende Lösung berechnen sich mit dem Modell von Fiserova-Bergerova et al. (1990) und dem Algorithmus des IH SkinPerm-Modells (Tibaldi et al. 2014) Fluxe von 7,1 bzw. 1,9 µg/cm^2^ und Stunde. Unter der Annahme einer einstündigen Exposition von 2000 cm^2^ Hautoberfläche (Standardbedingungen) würde dies Aufnahmemengen von 14,2 bzw. 3,8 mg entsprechen. Für die weitere Abschätzung wird von einer resorbierten Menge von 11 mg 1H-1,2,4-Triazol aus dem In-vitro-Test ausgegangen.

Bei 24-stündiger Applikation von 1 ml einer 9%igen wässrigen Formaldehydlösung auf Schweineohrhaut in Franz-Diffusionszellen (mit Parafilm abgedeckt, 0,79 cm^2^) mit phosphatgepufferter Kochsalzlösung im Rezeptorkompartiment wurde aus der wiedergefundenen Formaldehydmenge in der Haut und im Rezeptor ein Flux von 39,4 µg/cm^2^ und Stunde bestimmt. Der Permeationskoeffizient war 4 × 10^–4^ cm/h, die Verzögerungszeit betrug 7,7 h (López-Sánchez et al. 2021). Für eine 0,5%ige Lösung entspricht der Flux 2 µg/cm^2^ und Stunde, sodass unter Standardbedingungen 4 mg aufgenommen werden.

Ratten und Meerschweinchen erhielten (vermutlich offen) eine 1%ige wässrige Lösung von ^14^C-**Formaldehyd** (0,1 mg in 10 µl, 2 cm^2^) auf die Haut. Nach 72 Stunden wurden insgesamt 32 % der applizierten Radioaktivität (bei Ratten) und ca. 36 % der applizierten Radioaktivität (bei Meerschweinchen) in Urin, Faeces, Ausatemluft und Karkasse gefunden (ECHA 2022 b). Bei der Worst-Case-Annahme von 36 % Resorption einer 0,5%igen Lösung in einer Stunde, also 0,025 mg/cm^2^ (0,05 mg in 10 µl auf 2 cm^2^ wie oben) appliziert auf 2000 cm^2^ (50 mg), würden 18 mg aufgenommen werden. Mit dem Modell von Fiserova-Bergerova et al. (1990) und dem Algorithmus des IH SkinPerm-Modells (Tibaldi et al. 2014) errechnen sich unter Standardbedingungen Aufnahmemengen von 157 bzw. 33 mg.

## Tierexperimentelle Befunde und In-vitro-Untersuchungen

### Reproduktionstoxizität

In einer Screeningstudie nach OECD-Prüfrichtlinie 421 an Wistar-Hannover-Ratten wurden bis zur höchsten getesteten Dosis von 100 mg *N*,*N*-Bis(2-ethylhexyl)-((1,2,4-triazol-1-yl)methyl)amin/kg KG und Tag, gegeben per Schlundsonde, keine fetotoxischen Effekte festgestellt (Hartwig und MAK Commission 2017). Eine vollständige Untersuchung der Teratogenität ist bei dieser Prüfrichtlinie nicht vorgesehen.

Neue Daten liegen zu diesem Endpunkt nicht vor.

### Genotoxizität

An Bakterien und Säugerzellen wirkt *N*,*N*-Bis(2-ethylhexyl)-((1,2,4-triazol-1-yl)methyl)amin nicht mutagen. Ein Test auf Chromosomenaberrationen in Humanlymphozyten ergab nur unter Zugabe eines metabolischen Aktivierungssystems ein positives Ergebnis, das im Wiederholungsexperiment und in einem weiteren Chromosomenaberrationstest nicht bestätigt wurde. In zwei In-vivo-Mikronukleustests hatte der Stoff nach Gabe per Schlundsonde keine klastogene Wirkung im Knochenmark von Hamstern (Hartwig und MAK Commission 2017).

Neue Daten liegen zu diesem Endpunkt nicht vor.

### Kanzerogenität

Hierzu liegen keine Daten vor.

## Bewertung 

Das grundlegende Vorgehen zur Bewertung eines Arbeitsstoffes ist der MAK- und BAT-Werte-Liste zu entnehmen (DFG 2025).

Kritische Effekte sind die kanzerogene und lokal reizende Wirkung des Hydrolyseprodukts Formaldehyd.

**Krebserzeugende Wirkung. **Es liegen keine Untersuchungen der krebserzeugenden Wirkung von *N*,*N*-Bis(2-ethylhexyl)-((1,2,4-triazol-1-yl)methyl)amin vor. *N*,*N*-Bis(2-ethylhexyl)-((1,2,4-triazol-1-yl)methyl)amin weist in den vorliegenden Untersuchungen kein genotoxisches Potenzial auf, eine mögliche genotoxische Wirkung am wahrscheinlichen Zielgewebe des oberen Atemtrakts bzw. der Nase (wie bei Formaldehyd) ist jedoch nicht untersucht.

Wegen der sofortigen Hydrolyse wird davon ausgegangen, dass *N*,*N*-Bis(2-ethylhexyl)-((1,2,4-triazol-1-yl)methyl)amin beim Auftreffen im Atemtrakt sofort vollständig zu Formaldehyd, Bis(2-ethylhexylamin) und 1H-1,2,4-Triazol hydrolysiert und der Abbau des aus *N*,*N*-Bis(2-ethylhexyl)-((1,2,4-triazol-1-yl)methyl)amin entstehenden Formaldehyds langsamer erfolgt als dessen Bildung.

Die lokale Kanzerogenität des Hydrolyseprodukts Formaldehyd ist ausführlich dokumentiert (Greim 2000; Hartwig 2010). Formaldehyd ruft Nasentumoren hervor, jedoch erst bei Konzentrationen, die die Entgiftungskapazitäten des Nasengewebes überschreiten. Daher ist Formaldehyd in Kanzerogenitäts-Kategorie 4 eingestuft.

Aufgrund der lokalen kanzerogenen Wirkung von Formaldehyd könnte *N*,*N*-Bis(2-ethylhexyl)-((1,2,4-triazol-1-yl)methyl)amin in Analogie zu Formaldehyd in Kanzerogenitäts-Kategorie 4 eingestuft werden. Da jedoch kein MAK-Wert für *N*,*N*-Bis(2-ethylhexyl)-((1,2,4-triazol-1-yl)methyl)amin abgeleitet werden kann, wird der Stoff der Kanzerogenitäts-Kategorie 2 zugeordnet und erhält die Fußnote „Voraussetzung für Kategorie 4 prinzipiell erfüllt, aber Daten für MAK- oder BAT-Wert-Ableitung nicht ausreichend“.

**MAK-Wert. **Es liegen keine Inhalationsstudien mit *N*,*N*-Bis(2-ethylhexyl)-((1,2,4-triazol-1-yl)methyl)amin am Menschen oder am Tier vor, aus denen ein MAK-Wert abgeleitet werden kann.

*N*,*N*-Bis(2-ethylhexyl)-((1,2,4-triazol-1-yl)methyl)amin ist ein Formaldehydabspalter. Formaldehyd zeigt deutliche Reizwirkungen in der Nase und kann bei chronischer Exposition Tumoren im Epithel der Nasenschleimhaut erzeugen. Die Entgiftungskapazitäten des Nasengewebes für Formaldehyd werden bei einem MAK-Wert von 0,3 ml/m^3^ (0,37 mg/m^3^) nicht überschritten (Greim 2000; Hartwig 2010).

Der Dampfdruck von *N*,*N*-Bis(2-ethylhexyl)-((1,2,4-triazol-1-yl)methyl)amin (7,1 × 10^–5^ hPa) zeigt, dass der Stoff als Aerosol vorliegen kann. Für Aerosole ist durch die Impaktierung im Atemtrakt mit einer stärkeren Wirkung als die durch dampfförmigen Formaldehyd verursachte zu rechnen, wie mit einem anderen Formaldehydabspalter, zu dem eine Inhalationsstudie vorliegt, gezeigt wurde (siehe Begründung *N*,*N′*,*N′′*-Tris(β-hydroxyethyl)hexahydro-1,3,5-triazin; Hartwig und MAK Commission 2023).

Für *N*,*N*-Bis(2-ethylhexyl)-((1,2,4-triazol-1-yl)methyl)amin kann somit kein MAK-Wert festgesetzt werden. Eine Spitzenbegrenzung entfällt.

Bei Anwendung in verdünnten wässrigen Lösungen sollte mit einer vollständigen Hydrolyse gerechnet und daher der MAK-Wert für Formaldehyd (Greim 2000; Hartwig 2010) eingehalten werden.

**Fruchtschädigende Wirkung. **Da kein MAK-Wert abgeleitet wird, entfällt die Zuordnung zu einer risikobasierten Schwangerschaftsgruppe. Da weder valide Daten zur Entwicklungstoxizität noch Hinweise auf eine mögliche entwicklungstoxische Wirkung vorliegen, lässt sich ein entsprechender Verdacht (Gruppe B (Verdacht)) nicht begründen.

**Keimzellmutagene Wirkung. **Aus den vorliegenden Untersuchungen ergab sich für *N*,*N*-Bis(2-ethylhexyl)-((1,2,4-triazol-1-yl)methyl)amin kein Verdacht auf eine genotoxische Wirkung in somatischen Zellen (Hartwig und MAK Commission 2017). Neue Daten zur genotoxischen Wirkung oder Untersuchungen zur Wirkung auf die Keimzellen liegen nicht vor.

Wird davon ausgegangen, dass nach Inhalation durch Hydrolyse sofort das gesamte Formaldehyd im oberen Atemtrakt freigesetzt wird, ist dieses wahrscheinlich nicht systemisch verfügbar. Daten dazu fehlen jedoch.

Theoretisch könnte *N*,*N*-Bis(2-ethylhexyl)-((1,2,4-triazol-1-yl)methyl)amin in Analogie zu Formaldehyd in die Kategorie 5 für Keimzellmutagene (Greim 2000) eingestuft werden, jedoch kann für *N*,*N*-Bis(2-ethylhexyl)-((1,2,4-triazol-1-yl)methyl)amin kein MAK-Wert aufgestellt werden.

Da Daten zur systemischen Bioverfügbarkeit von *N*,*N*-Bis(2-ethylhexyl)-((1,2,4-triazol-1-yl)methyl)amin und dem durch Hydrolyse freigesetzten Formaldehyd fehlen, liegt kein experimenteller Beleg vor, dass der freigesetzte Formaldehyd in aktiver Form die Keimzellen erreicht. Daher wird *N*,*N*-Bis(2-ethylhexyl)-((1,2,4-triazol-1-yl)methyl)amin in Kategorie 3 B für Keimzellmutagene eingestuft.

**Hautresorption. **Der Stoff hydrolysiert in Wasser sofort zu Bis(2-ethylhexylamin), 1H-1,2,4-Triazol und Formaldehyd. Der Stoff ist ätzend und es liegen keine Daten zur Hautresorption vor. Wegen der sofortigen Hydrolyse wird die Hautresorption der Hydrolyseprodukte bei einer nicht mehr als reizend angesehenen Konzentration von 0,5 % bewertet.

**Bis(2-ethylhexylamin)** ist ätzend und es gibt keine Studien zur Hautresorption und zur Toxizität nach wiederholter dermaler Gabe. Die dermale LD_50_ liegt bei 958 mg/kg KG bei Kaninchen. Hierbei ist zu berücksichtigen, dass es aufgrund der ätzenden Wirkung zu einer Zerstörung der Hautbarriere und dadurch zu einer erhöhten Aufnahme über die Haut gekommen sein kann (ECHA 2023). Diese Studie kann daher keine Markierung mit „H“ begründen. Eine Berechnung mit den Modellen ist nicht möglich, da der log K_OW_ > 6 ist (ECHA 2023) und damit außerhalb des Gültigkeitsbereichs der Modelle liegt.

Für die Bewertung der Hautresorption von **1H-1,2,4-Triazol** wird von einer resorbierten Menge von 11 mg unter Standardbedingungen ausgegangen. Der orale systemische subchronische NOAEL beträgt bei Ratten 37,8 mg/kg KG (90-Tage-Studie ähnlich wie OECD-Prüfrichtlinie 408; ECHA 2022 a). Zur toxikokinetischen Übertragung dieser Dosis als systemischen NOAEL auf den Menschen werden berücksichtigt: der dem toxikokinetischen Unterschied zwischen Ratte und dem Menschen entsprechende speziesspezifische Korrekturwert (1:4), die experimentelle orale Resorption von 90 %, die tägliche Exposition der Tiere im Vergleich zur fünftägigen Exposition pro Woche am Arbeitsplatz (7:5), das Körpergewicht (70 kg) des Menschen, eine mögliche Wirkungsverstärkung mit der Zeit (1:2) und die Übertragung der Daten des Tierversuchs auf den Menschen (1:2). Daraus errechnet sich eine systemisch tolerable Menge von 209 mg (37,8 mg/kg × 70 kg / 4 × 7 / 5 / 2 / 2 × 0,9). Damit liegt die Aufnahme über die Haut bei weniger als 25 % der systemisch tolerablen Menge.

Ratten und Meerschweinchen erhielten (vermutlich offen) eine 1%ige wässrige Lösung ^14^C-**Formaldehyd** (0,1 mg in 10 µl, 2 cm^2^) auf die Haut. Nach 72 Stunden wurden insgesamt 32 % (Ratten) und ca. 36 % (Meerschweinchen) in Urin, Faeces, Ausatemluft und Karkasse gefunden (ECHA 2022 b). Bei der Worst-Case-Annahme von 36 % Resorption einer 0,5%igen Lösung in einer Stunde, also 0,025 mg/cm^2^ (0,05 mg in 10 µl auf 2 cm^2^ wie oben) appliziert auf 2000 cm^2^ (50 mg), werden 18 mg aufgenommen. Mit dem Modell von Fiserova-Bergerova et al. (1990) und dem Algorithmus des IH SkinPerm-Modells (Tibaldi et al. 2014) berechnen sich 157 bzw. 33 mg aufgenommene Menge in einer Stunde. Mit dem In-vitro-Versuch an Schweineohrhaut würde sich eine aufgenommene Menge von 4 mg ergeben (López-Sánchez et al. 2021). Unter Annahme einer konstanten Resorptionsrate und, im Sinne einer Worst-Case-Betrachtung, raschen Hydrolyse des *N*,*N*-Bis(2-ethylhexyl)-((1,2,4-triazol-1-yl)methyl)amin werden demnach bei 157 mg maximal 3,27 mg Formaldehyd/1,25 min (mittlere Halbwertszeit des Formaldehyds im Blut; Kaden et al. 2010) resorbiert bzw. freigesetzt. Die transdermale Aufnahme in den letzten sechs Halbwertszeit-Intervallen führt somit im Fließgleichgewicht zu einer im Blut zirkulierenden Formaldehydmenge von (3,3 + 1,6 + 0,8 + 0,4 + 0,2 + 0,1) mg = 6,4 mg. Der physiologisch bedingte Formaldehydspiegel im Blut des Menschen beträgt etwa 2–3 mg/l (10–15 mg in 5 l Blut, frei und reversibel gebunden) (Heck et al. 1985).

*N*,*N*-Bis(2-ethylhexyl)-((1,2,4-triazol-1-yl)methyl)amin wird weiterhin nicht mit „H“ markiert, da die Abschätzung für Formaldehyd nach Fiserova-Bergerova et al. (1990) deutlich höher ist als die aus In-vivo-Daten, aus In-vitro-Daten und aus der IH SkinPerm-Modellierung, nach denen die Erhöhung der Formaldehyd-Gesamtmenge im Blut deutlich im Variationsbereich der physiologischen Belastung liegt. Für die anderen beiden Hydrolyseprodukte fehlen Daten bzw. beträgt die Aufnahme über die Haut weniger als 25 % der systemisch tolerablen Menge.

**Sensibilisierende Wirkung. **Es liegen weiterhin keine Daten zur hautsensibilisierenden Wirkung von *N*,*N*-Bis(2-ethylhexyl)-((1,2,4-triazol-1-yl)methyl)amin beim Menschen vor. Die Ergebnisse eines modifizierten Maximierungstests und eines nach Prüfrichtlinie durchgeführten Maximierungstests lassen aber eindeutig ein hautsensibilisierendes Potenzial erkennen (Hartwig und MAK Commission 2017). Auch aufgrund der Eigenschaft der Substanz, Formaldehyd freizusetzen, bleibt *N*,*N*-Bis(2-ethylhexyl)-((1,2,4-triazol-1-yl)methyl)amin weiterhin mit „Sh“ markiert.

Zur atemwegssensibilisierenden Wirkung liegen ebenfalls keine Daten vor, daher erfolgt keine Markierung mit „Sa“.
